# Supplementary material for: Neural network based prediction of the efficacy of ball milling to separate cable waste materials
Source: Commun Eng. 2023 May 19;2:27. doi: 10.1038/s44172-023-00079-y (PMC10956031; doi:10.1038/s44172-023-00079-y)
Supplement: Supplementary file 2 — Supplementary Information [file 44172_2023_79_MOESM2_ESM.pdf]

Supplementary Information

Neural network based prediction of the efficacy of ball milling to separate cable waste materials

Jiaqi Lu [a, b, *], *Mengqi Han* [b], *Shogo Kumagai* [a, *], *Guanghui Li* [b], *Toshiaki Yoshioka* [a]

[a] Graduate School of Environmental Studies, Tohoku University, 6-6-07 Aoba, Aramaki-aza, Aoba-ku, Sendai, Miyagi 980-8579, Japan

[b] Innovation Centre for Environment and Resources, Shanghai University of Engineering Science, No.333 Longteng Road, Songjiang District, Shanghai 201620, China

[*] Corresponding author.

Jiaqi Lu, wilsherelu@foxmail.com, Tel: +86-21-67795965

Shogo Kumagai, kumagai@tohoku.ac.jp, Tel: +81-22-795-7212

Supplementary Notes 1 Introduction of Cu and PVC recovery from cable waste by ball milling

As shown in Figure S1, the developed separation process for cable waste first involved de-plasticizing the PVC cover using organic solvents (such as diethyl ether), which can dissolve plasticizers but not PVC [1, 2]. Cables was put into a Soxhlet-extractor filled by 150 mL of diethyl ether, which were heated at 75 ºC. Soxhlet-extraction was carried out for the maximum 300 min. The same number of cables were immersed in diethyl ether at room temperature for a maximum of 60 minutes in order to create a sample with a low extraction yield. Flexible electric cables become brittle by extracting plasticizers from covering PVC, and the de-plasticized PVC coverings can be crushed into scraps by ball milling and separated from the Cu core.

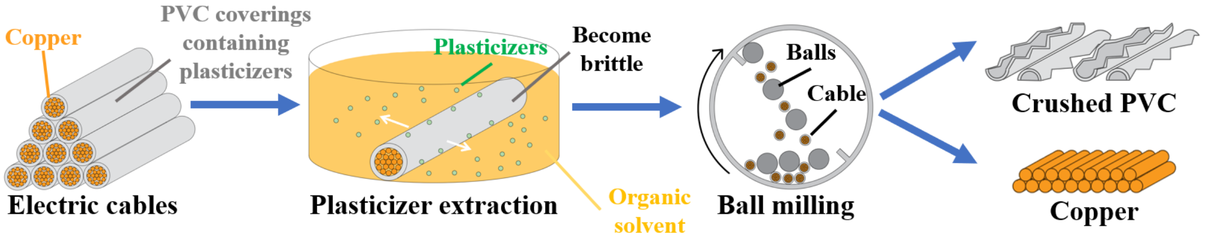

Figure S1. Schematic diagram of Cu and PVC recovery from the separation of de-plasticized cables by ball milling.

The ball milling treatment of de-plasticized cables with different Y_{ext} was carried out using a stainless-steel ball mill reactor made by (15cm in diameter, PM-001, AS ONE Co., Japan)) with tungsten carbide balls (AS ONE Co., Japan). The grinding balls have diameters of 10, 15, and 20 mm and weigh 7.8, 26.0, and 62.0 g/ball, respectively. In order to assess the size distribution of the crushed samples, the crushed cables were divided into 12 parts between 100 m and 4.75 mm using an electromagnetic sieve shaker (3000/min vibration, 1.8mm

amplitude, AS200Basic, AS ONE Co., Japan). Separation yield (Y_{sep}) of cable was defined by Equation (S1):

$$Y_{sep} = \frac{m_s}{m_0} \times 100\% \tag{S1}$$

where m_s [g] is the amount of separated cable; m_0 [g] is the amount of all the cables after ball milling.

Supplementary Notes 2 Mechanism of cable separation by ball milling

Based on our previous experimental investigation and analysis by discrete element method [2], the mechanism of cable separation by ball milling is depicted in Figure S2. It was found that the cable hardness increased and its elasticity reduced when the plasticizer was removed from the flexible PVC covering. Cracks from the edge to the center of the PVC covering can be formed due to inelastic collisions between the balls and cables. Thicker cables need more impact energy to create a crack. As the number of cracks accumulates, the Cu wrapped in the cables can slide out from the crushed PVC covering. Longer cables and larger numbers of cables will increase the required more time to accumulate cracks before complete separation. Because kinetic energy is conserved during elastic collisions, the impact energy from small balls cannot be utilized for crushing cables as it only imparts kinetic energy to the other balls and cables. Despite the greater impact energy from larger ball sizes and higher rotational speeds than moderate conditions, the increased impact energy will be partially wasted; thus, the time efficiency cannot be improved in this way. In addition, unnecessary collisions on separated PVC and Cu may produce finer particles, which will degrade the separation accuracy. In summary, moderate conditions of ball size, moderate rotational speed, and shorter cable lengths provide benefits for crushing a specific type and diameter of cables.

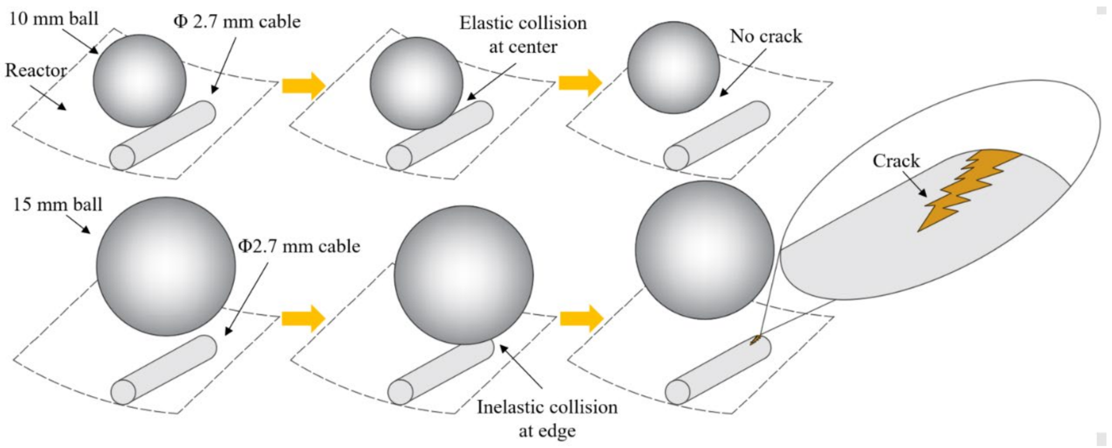

Figure S2[2]. Schematic diagram showing the mechanism of crushing cables by ball milling. (reuse permitted by Elsevier, License Number: 5393470579376)

Table S1. Literature review on the recent advance in the material recycling by cable separation.

Separation mechanism	Recycled materials	Max separation yield [%]	Process modeling	Highlight	Reference number
Plasticizer extraction and ball milling	Cu, PVC	100	Discrete element method	>99% of Cu purity for thin cable separation	1, 2
Cu slipping from the swollen cable	Cu, PVC	100	Discrete element method and computation fluid dynamics	Complete and perfect separation with the revealed mechanism based on the simulation	3, 4
Combined cable swelling and ball milling	Cu, PVC	100	None	The application for real cable waste with solvent regeneration test	5
Gravity, electrostatic	Al, PVC	99	None	Process selection by multiple criteria decision making	6
Comminution followed by electrostatic	Al	91	Regression plane	The impact of grain size from comminution on electrostatic separation	7
Electrostatic	Cu	95	Two-factor quadric polynomial model	Efficient separation for a fine PVC/Cu granular mixture	8
Comminution followed by sieving or magnetic and electrostatic	Al, Cu-clad steel	99	None	Detailed mass balance data and economic assessment for each process	9
Comminution and sieving followed by electrostatic	Al, Cu-clad steel	94	None	Comparison of plate-type and screen-type electrostatic separators	10
Froth flotation	PVC, PE, metals	90	None	High-purity recycled PVC verified by differential scanning calorimetry and	11

				thermogravimetric analysis	
Jigging	PE	88	None	Separation of regular PE and cross-linked PE	12
Jigging, shaking, froth flotation	Cu, plastic	97	None	Froth flotation was not as effective as jigging and shaking	13
Mechanical followed by spouted bed elutriation	Steel-coated Cu, Al, PVC, PE	90	None	Characterization of the recycled material	14
Microwave-assisted extraction	PVC	50	Support vector regression	100% removal of heavy metals from recycled PVC	15

Supplementary Notes 3 The quantitative relation between *IM* and ball diameter

In our previous study, the *IM* was calculated based on the simulation of ball milling process including the motions of grinding balls and charged cables. The simulation was carried out by a discrete element method in Multiphase Flow with Interphase eXchanges [16, 17]. During the simulation, the impact energy was calculated according to the collision intensity between the grinding balls and cables as follows:

$$IM = \sum \frac{1}{2} m v_{bc}^{2} \tag{S2}$$

where *IM* [J/s] is the sum of impact energy of the ball-to-cable collisions per second; *m* [g] is the mass of the grinding balls; and v_{bc} [m/s] represents the relative velocity between the ball and cable at the time of the collision. When the size of mill and other mechanical conditions are fixed, a linear relation can be assumed between *IM* and *m*. In consequence, a cubic function can be used to model *IM* by the ball diameter (*d*) as shown in Equation (S3).

$$IM = a \times d^3 \tag{S3}$$

Through the curve fitting based on the *d* vs. *IM* for different types of cables, the quantitative relation can be derived in Figure S3.

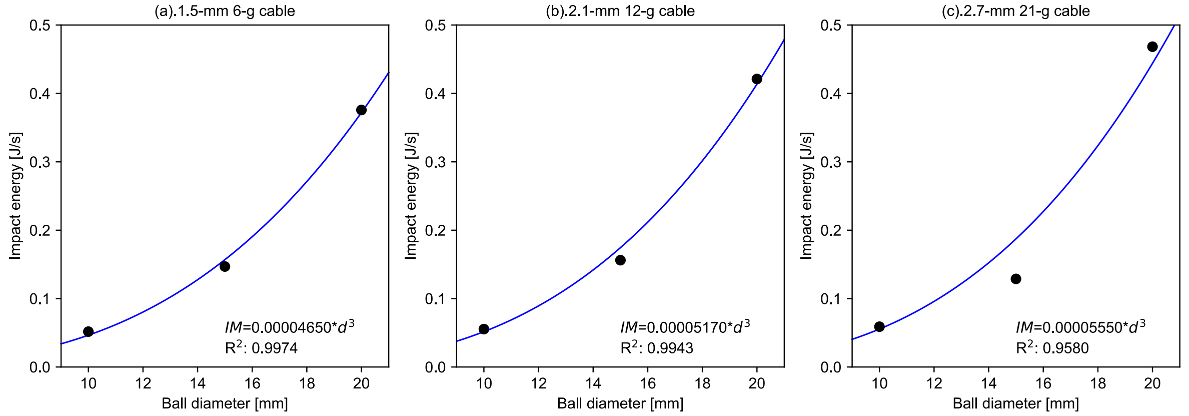

Figure S3. Fitted cubic relation between ball diameter (*d*) and impact energy (*IM*) for different types of cables: (a) 1.5-mm 6-g cable; (b) 2.1-mm 12-g cable; (c) 2.7-mm 21-g cable.

Supplementary Notes 4 Model details of NN_k and NN_c

The detailed structures, including the input, output, and activation functions of each layer, of NN_k and NN_c are shown in Figure S4. Along with the origin values of normalized input features, an exponential function was applied to add the non-linearity of input features both for NN_k and NN_c. To calculate *k*, the old Y_{sep} at previous running time is also included in the calculation; meanwhile, the accumulation of impact energy (*IM*) is considered by $\log_{10}(\frac{1}{IM \cdot t})$ for quantifying *c*. The number of layer parameters can be found in Table S2.

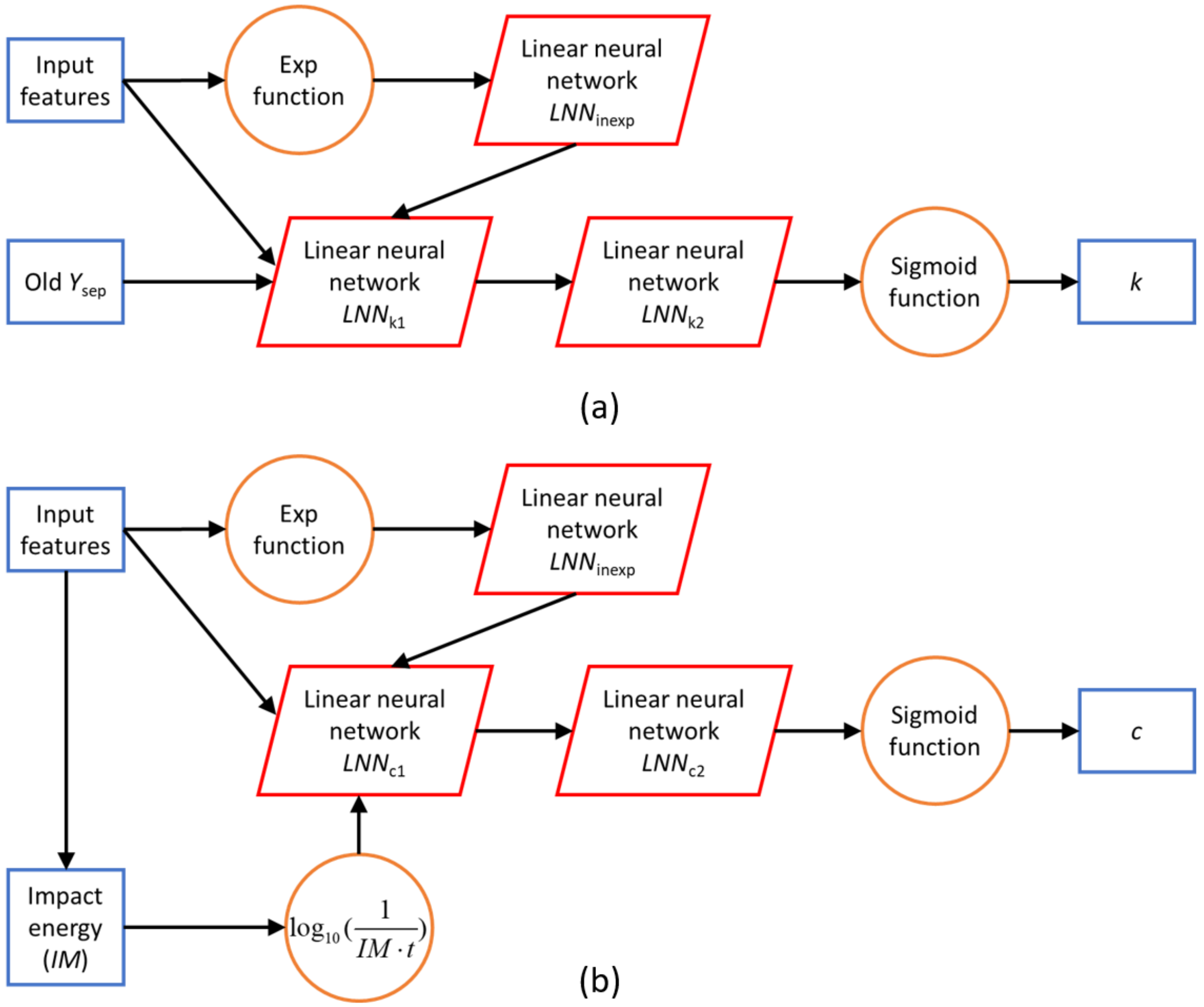

Figure S4. Network structures including input, output, hidden layers, and activations functions of NN_k (a) and NN_c (b).

Table S2. The properties of each hidden layers in NN_k and NN_c.

Name of hidden layer	Input number of feature dimension	Output number of feature dimension
LNN_{inexp}	5	1
LNN_{k1}	7	10
LNN_{k2}	10	1
LNN_{c1}	7	10
LNN_{c2}	10	1

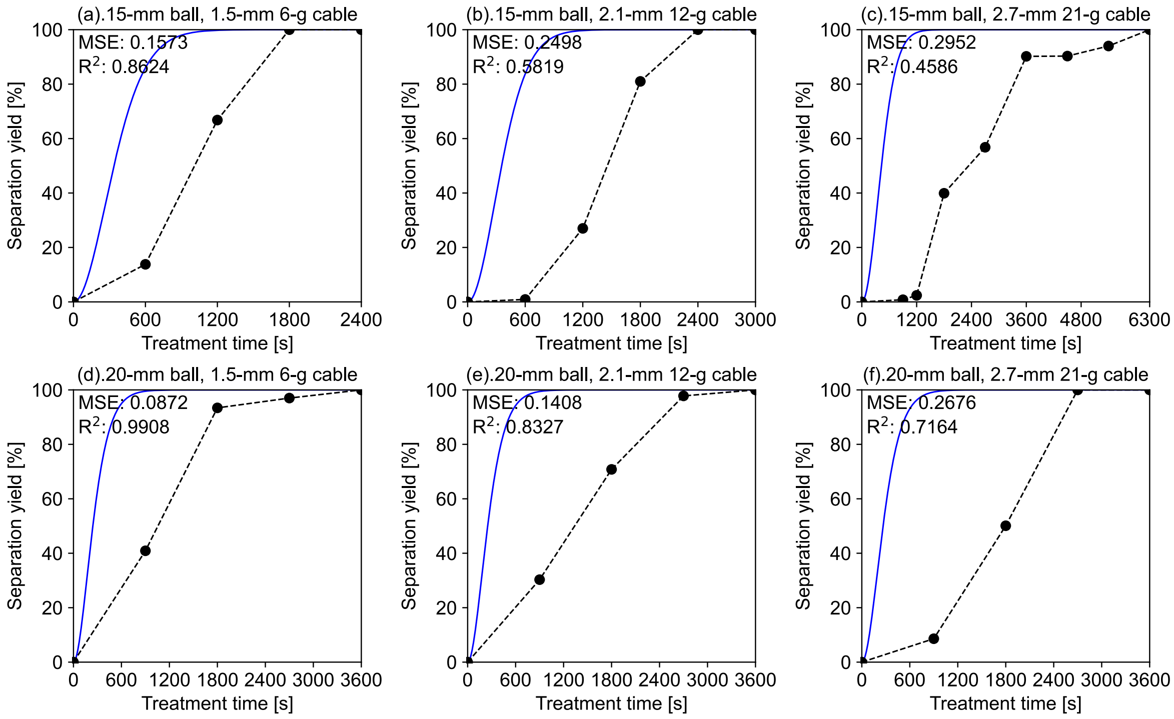

Figure S5. $Y_{\text{sep-exp}}$ and the predicted Y_{sep} for all experimental conditions (a-f) based on the trained model with 1 timestep. (the original training data and validation data can be found in at https://github.com/wilsherelu/Cable-separation-prediction.)

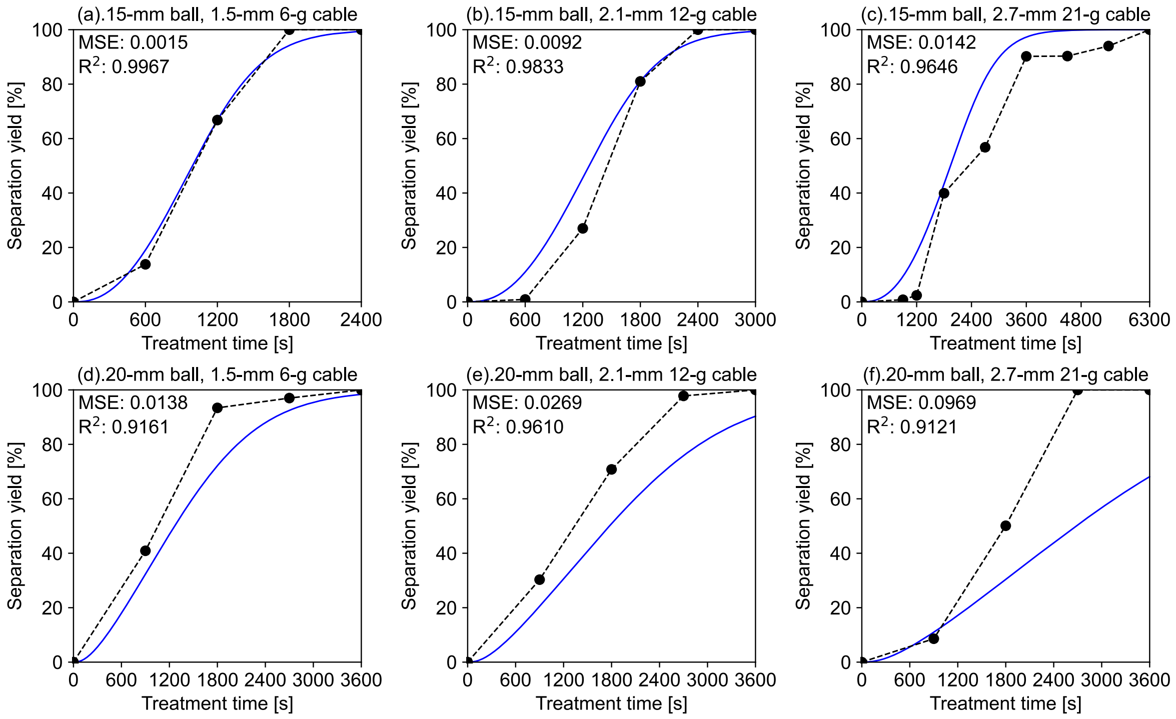

Figure S6. $Y_{\text{sep-exp}}$ and the predicted Y_{sep} for all experimental conditions (a-f) based on the trained model with 3 timesteps. (the original training data and validation data can be found in at https://github.com/wilsherelu/Cable-separation-prediction.)

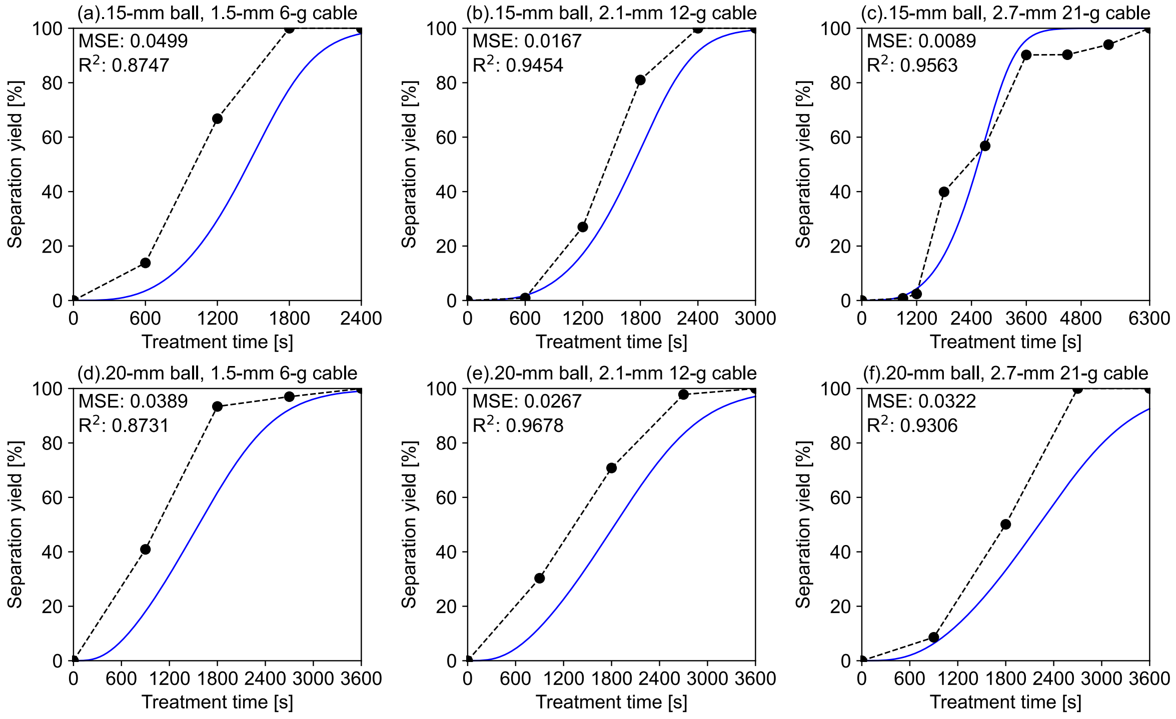

Figure S7. $Y_{\text{sep-exp}}$ and the predicted Y_{sep} for all experimental conditions (a-f) based on the trained model with 5 timesteps. (the original training data and validation data can be found in at https://github.com/wilsherelu/Cable-separation-prediction.)

Supplementary Reference

1. Xu J., Tazawa N., Kumagai S., Kameda T., Saito Y., Yoshioka T. Simultaneous recovery of high-purity copper and polyvinyl chloride from thin electric cables by plasticizer extraction and ball milling. *RSC Adv.* **8**, 6893-6903 (2018).

2. Xu J*., et al.* Validation of a deplasticizer–ball milling method for separating Cu and PVC from thin electric cables: A simulation and experimental approach. *Waste Manag.* **82**, 220-230 (2018).

3. Xu J*., et al.* Separation of copper and polyvinyl chloride from thin waste electric cables: A combined PVC-swelling and centrifugal approach. *Waste Manag.* **89**, 27-36 (2019).

4. Lu J., Xu J., Kumagai S., Kameda T., Saito Y., Yoshioka T. Separation mechanism of polyvinyl chloride and copper components from swollen electric cables by mechanical agitation. *Waste Manag.* **93**, 54-62 (2019).

5. Kumar H., Kumagai S., Kameda T., Saito Y., Yoshioka T. Highly efficient recovery of high-purity Cu, PVC, and phthalate plasticizer from waste wire harnesses through PVC swelling and rod milling. *React. Chem. Eng.* **5**, 1805-1813 (2020).

6. Sokolović J., Stanujkić D., Štirbanović Z. Selection of process for aluminium separation from waste cables by TOPSIS and WASPAS methods. *Miner. Eng.* **173**, 107186 (2021).

7. Bedeković G., Trbović R. Electrostatic separation of aluminium from residue of electric cables recycling process. *Waste Manag.* **108**, 21-27 (2020).

8. Catinean A., Dascalescu L., Lungu M., Dumitran L. M., Samuila A. Improving the recovery of copper from electric cable waste derived from automotive industry by corona-electrostatic separation. *Part. Sci. Technol.* **39**, 449-456 (2021).

9. Martins T. R., Mrozinski N. S., Bertuol D. A., Tanabe E. H. Recovery of copper and aluminium from coaxial cable wastes using comparative mechanical processes analysis. *Environ. Technol.* **42**, 3205-3217 (2021).

10. Martins T. R., Bertuol D. A., Tanabe E. H. Recovery of metals and polymers from coaxial cables using different configurations of electrostatic separators. *J. Mater. Cycles Waste Manag.* **24**, 633-641 (2022).

11. Barbakadze K., Brostow W., Granowski G., Hnatchuk N., Lohse S., Osmanson A. T. Separation of metal and plastic wastes from wire and cable manufacturing for effective recycling. *Resour. Conserv. Recycl.* **139**, 251-258 (2018).

12. Ito M., *et al.* Development of the reverse hybrid jig: Separation of polyethylene and cross-linked polyethylene from eco-cable wire. *Miner. Eng.* **174**, 107241 (2021).

13. Pita F., Castilho A. Separation of Copper from Electric Cable Waste Based on Mineral Processing Methods: A Case Study. *Minerals* **8**, 517 (2018).

14. Tanabe E. H., Silva R. M., Oliveira Júnior D. L., Bertuol D. A. Recovery of valuable metals from waste cables by employing mechanical processing followed by spouted bed elutriation. *Particuology* **45**, 74-80 (2019).

15. Jia C., Das P., Zeng Q., Gabriel J.-C. P., Tay C. Y., Lee J.-M. Activated recovery of PVC from contaminated waste extension cord-cable using a weak acid. *Chemosphere* **303**, 134878 (2022).

16. Mishra B., Rajamani R. K. The discrete element method for the simulation of ball mills. *Appl. Math. Model.* **16**, 598-604 (1992).

17. Garg R., Galvin J., Li T., Pannala S. Open-source MFIX-DEM software for gas–solids flows: Part I—Verification studies. *Powder Technol.* **220**, 122-137 (2012).
